# Supplementary material for: A molecular mechanism for the generation of ligand-dependent differential outputs by the epidermal growth factor receptor
Source: eLife. 2021 Nov 30;10:e73218. doi: 10.7554/eLife.73218 (PMC8716103; doi:10.7554/eLife.73218)
Supplement: Figure 3—source data 1. [file elife-73218-fig3-data1.doc]

**Figure 3-source data 1**

**Statistics of cryo-EM data collection and structure determination**

|  | EGFR(WT)  :EGF  juxtaposed | EGFR(WT)  :EGF  separated | EGFR(WT)  :TGF-α  juxtaposed | EGFR(WT)  :TGF-α  separated | EGFR(L834R)  :EGF  juxtaposed | EGFR(L834R)  :EGF  separated |
| --- | --- | --- | --- | --- | --- | --- |
| **Data collection** |  |  |  |  |  |  |
| Electron Microscope | Titan Krios | Titan Krios | Titan Krios | Titan Krios | Titan Krios | Titan Krios |
| Camera | Gatan K3 | Gatan K3 | Gatan K3 | Gatan K3 | Gatan K3 | Gatan K3 |
| Voltage (kV) | 300 | 300 | 300 | 300 | 300 | 300 |
| Magnification | 81,000 | 81,000 | 81,000 | 81,000 | 81,000 | 81,000 |
| Pixel size (Å) | 0.54 | 0.54 | 0.54 | 0.54 | 0.54 | 0.54 |
| Nominal does (e–/Å^2^) | 50 | 50 | 50 | 50 | 50 | 50 |
| Tilt angle (^o^) | 40 | 40 | 40 | 40 | 40 | 40 |
| Defocus range (μm) | -0.75 to -1.5 | -0.75 to -1.5 | -0.75 to -1.5 | -0.75 to -1.5 | -0.75 to -1.5 | -0.75 to -1.5 |
|  |  |  |  |  |  |  |
| **Image Processing** |  |  |  |  |  |  |
| Micrographs | 4,331 | 4,331 | 7,406 | 7,406 | 5,013 | 5,013 |
| Final particle number | 105,728 | 89,757 | 126,471 | 102,206 | 123,670 | 100,615 |
| Map resolution (Å) | 3.1 | 3.3 | 3.4 | 3.6 | 3.3 | 3.4 |
| Map sharpening *B* factor | -97.6 | -94.7 | -108.2 | -108.2 | -103.5 | -92.8 |
|  |  |  |  |  |  |  |
| **Model Refinement** |  |  |  |  |  |  |
| Rms deviation bonds (Å) | 0.007 | 0.006 | 0.004 | 0.007 | 0.004 | 0.004 |
| Rms deviation angles (^o^) | 0.691 | 0.732 | 0.639 | 0.820 | 0.663 | 0.705 |
| Map CC (mask) | 0.82 | 0.76 | 0.79 | 0.74 | 0.81 | 0.72 |
|  |  |  |  |  |  |  |
| **Validation** |  |  |  |  |  |  |
| Molprobility score | 1.96 | 2.20 | 2.05 | 2.45 | 1.91 | 2.19 |
| Clash score | 7.50 | 11.14 | 8.84 | 17.78 | 6.41 | 10.87 |
| Rotamer outliers (%) | 0.09 | 0.19 | 0.00 | 0.38 | 0.00 | 0.00 |
| Cβ outliers (%) | 0.00 | 0.00 | 0.00 | 0.00 | 0.00 | 0.00 |
| Ramachandran plot |  |  |  |  |  |  |
| Favored (%) | 90.08 | 86.00 | 88.97 | 82.75 | 89.55 | 86.14 |
| Allowed (%) | 9.92 | 14.00 | 11.03 | 17.25 | 10.45 | 13.86 |
