# Supplementary figures and images for: A molecular mechanism for the generation of ligand-dependent differential outputs by the epidermal growth factor receptor
Source: eLife. 2021 Nov 30;10:e73218. doi: 10.7554/eLife.73218 (PMC8716103; doi:10.7554/eLife.73218)

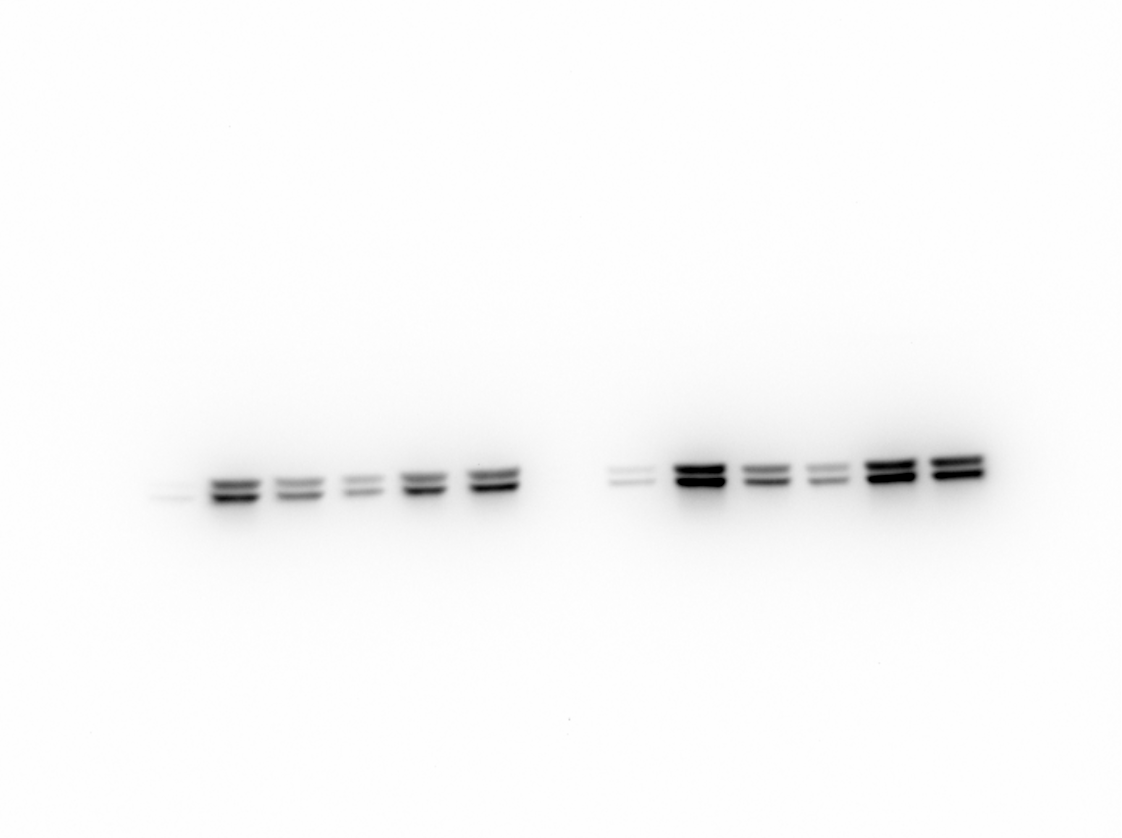

Supplement: Figure 9—source data 1. [file elife-73218-fig9-data1.tif]

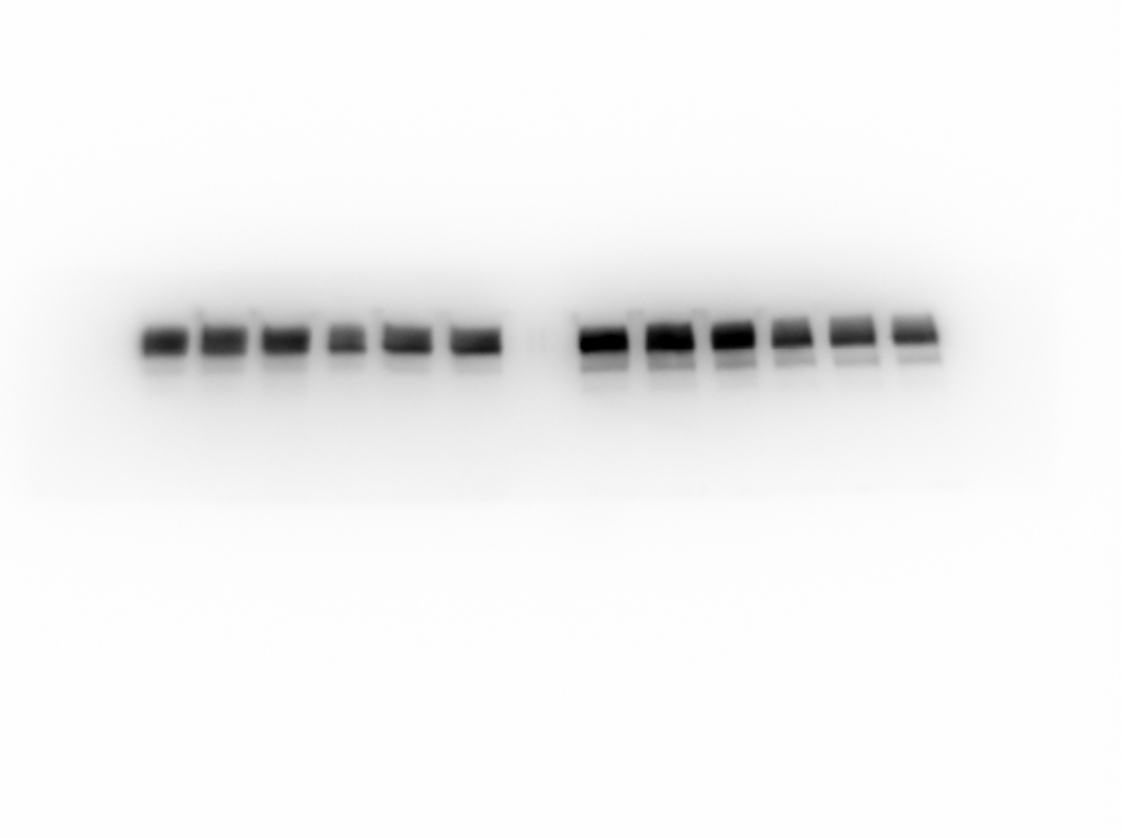

Supplement: Figure 9—source data 2. [file elife-73218-fig9-data2.tif]

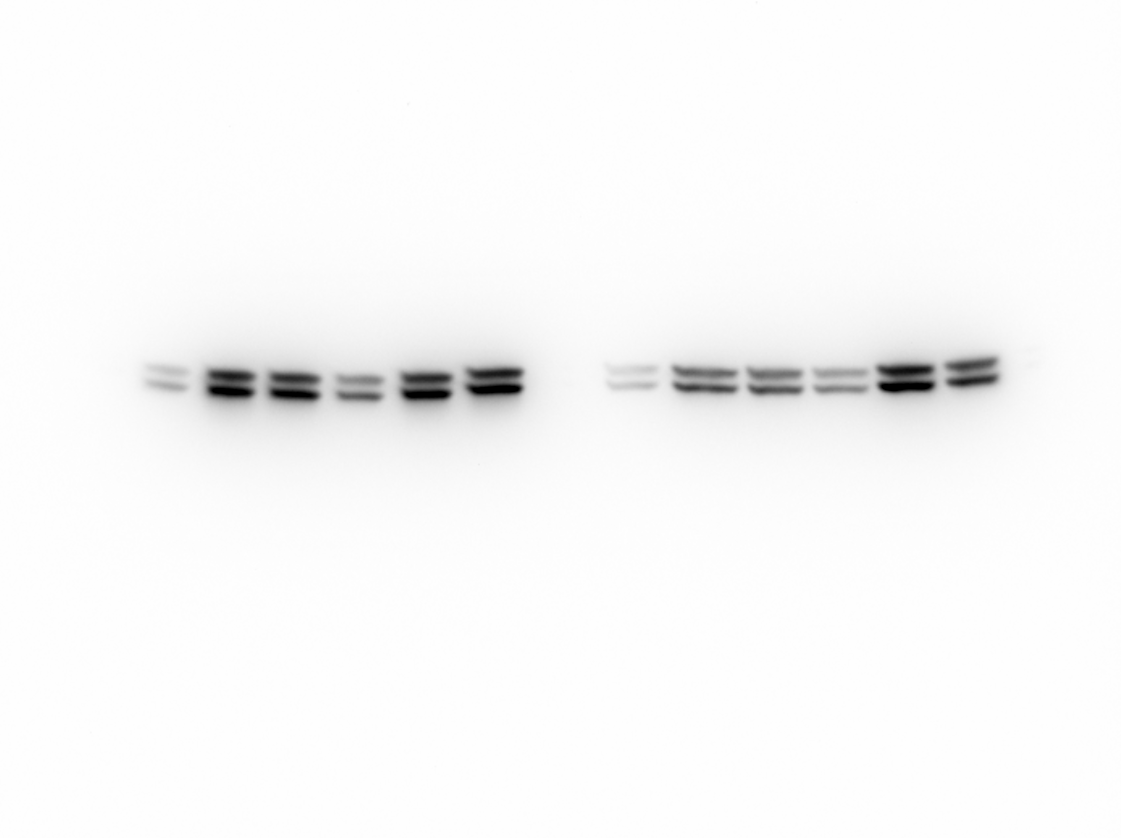

Supplement: Figure 9—source data 3. [file elife-73218-fig9-data3.tif]

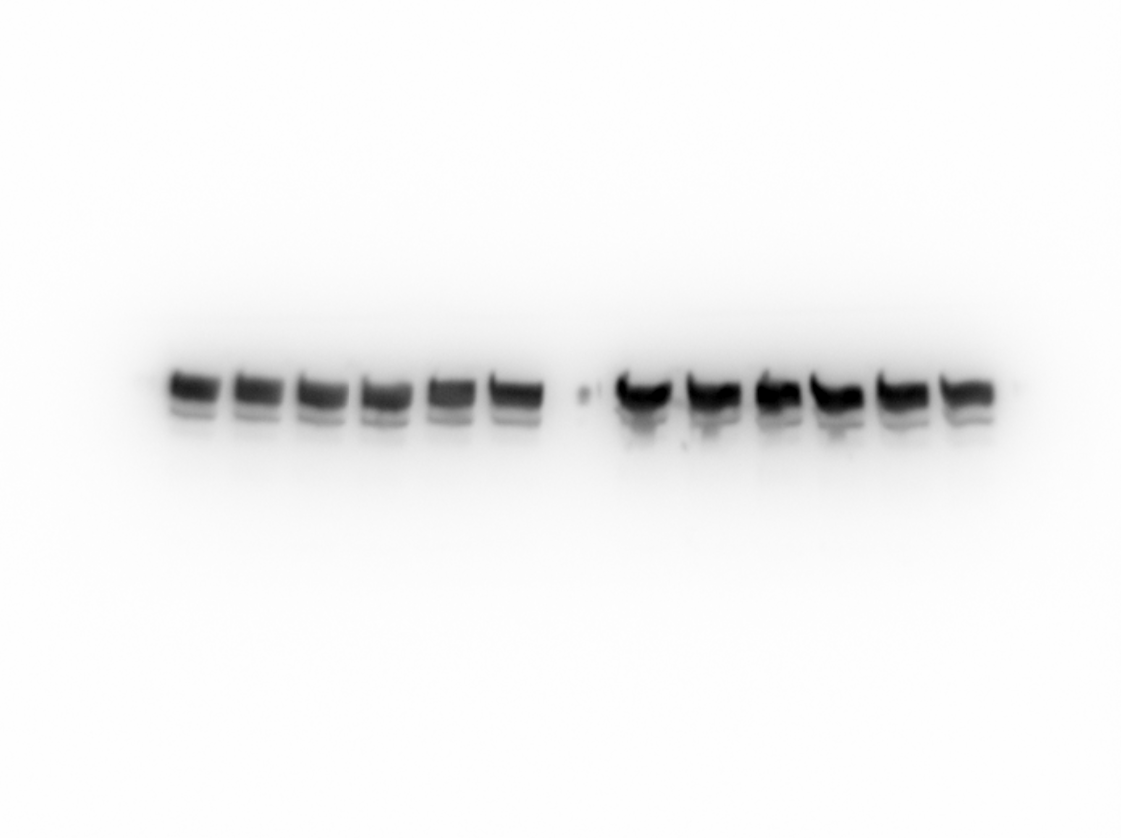

Supplement: Figure 9—source data 4. [file elife-73218-fig9-data4.tif]

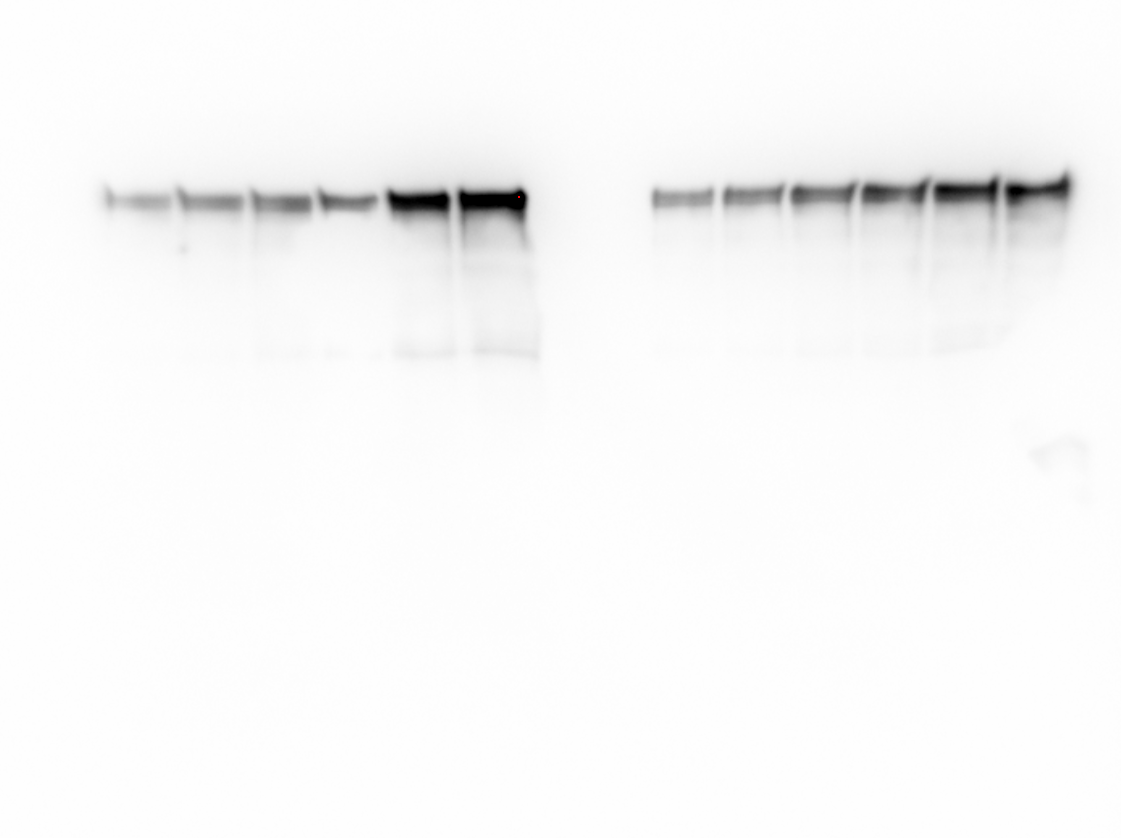

Supplement: Figure 9—figure supplement 1—source data 1. [file elife-73218-fig9-figsupp1-data1.tif]

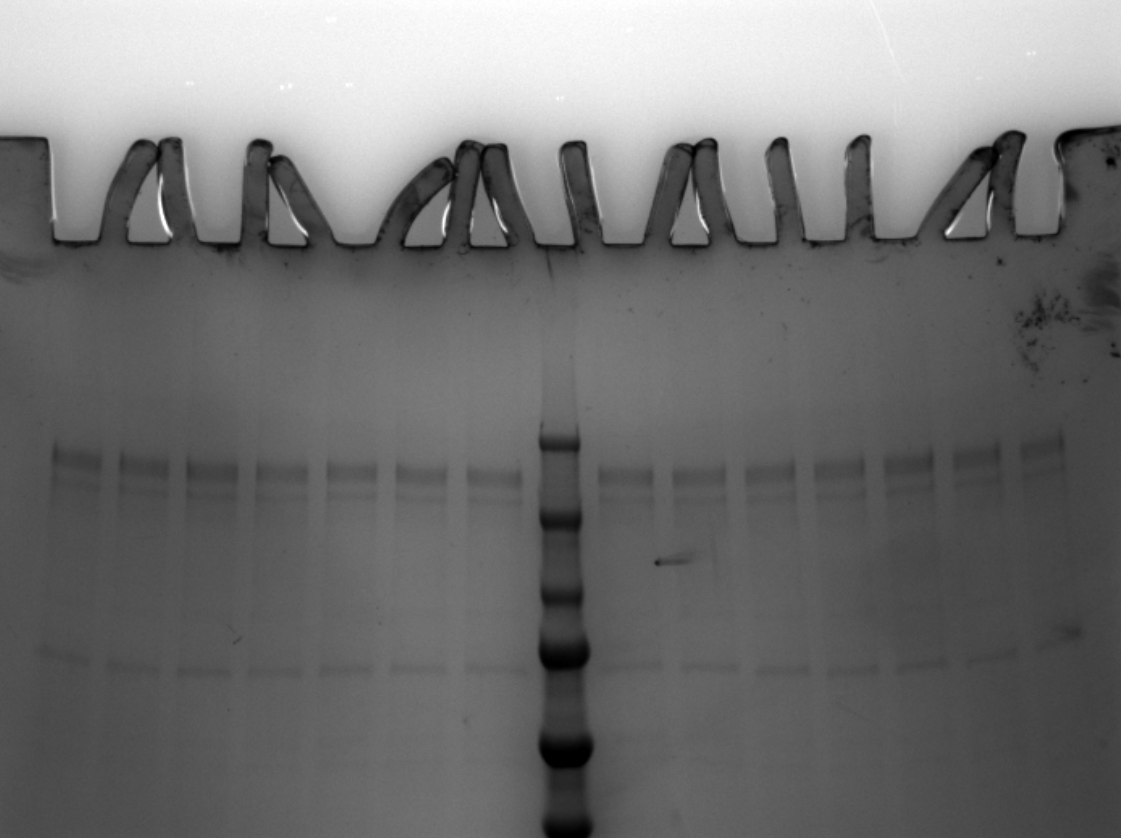

Supplement: Figure 9—figure supplement 1—source data 2. [file elife-73218-fig9-figsupp1-data2.tif]

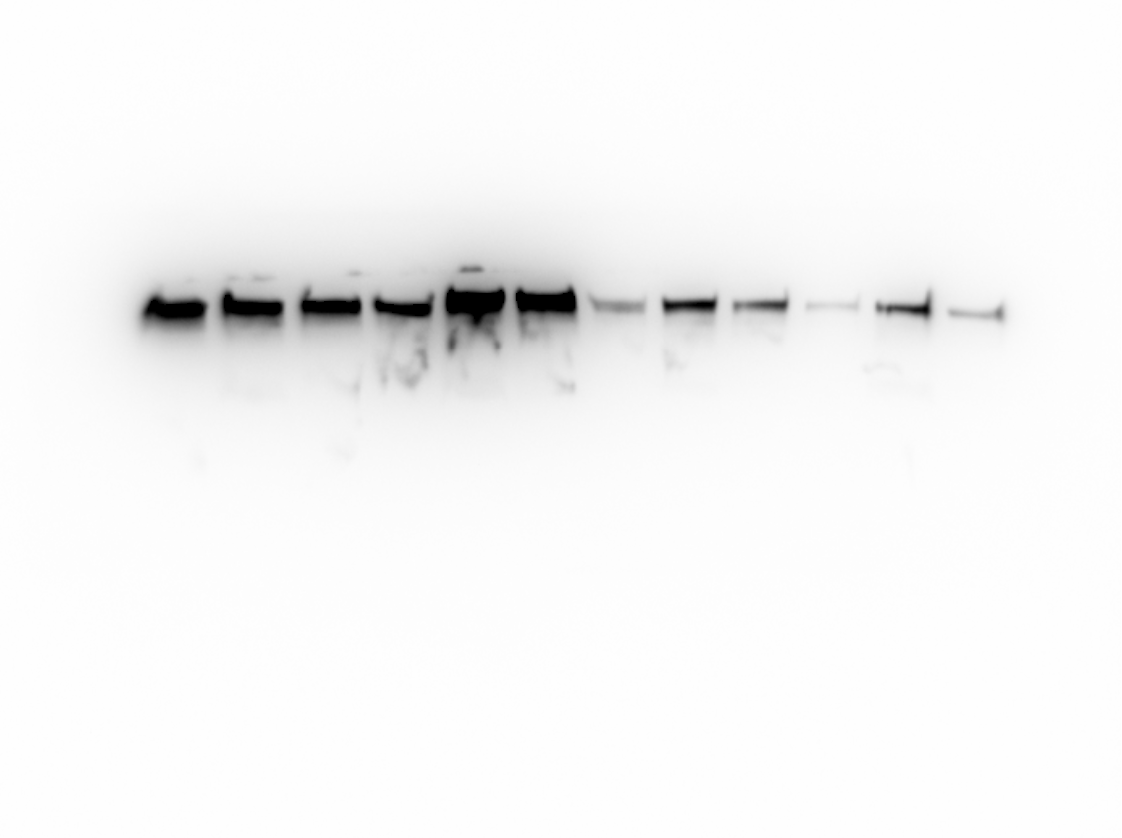

Supplement: Figure 9—figure supplement 1—source data 3. [file elife-73218-fig9-figsupp1-data3.tif]

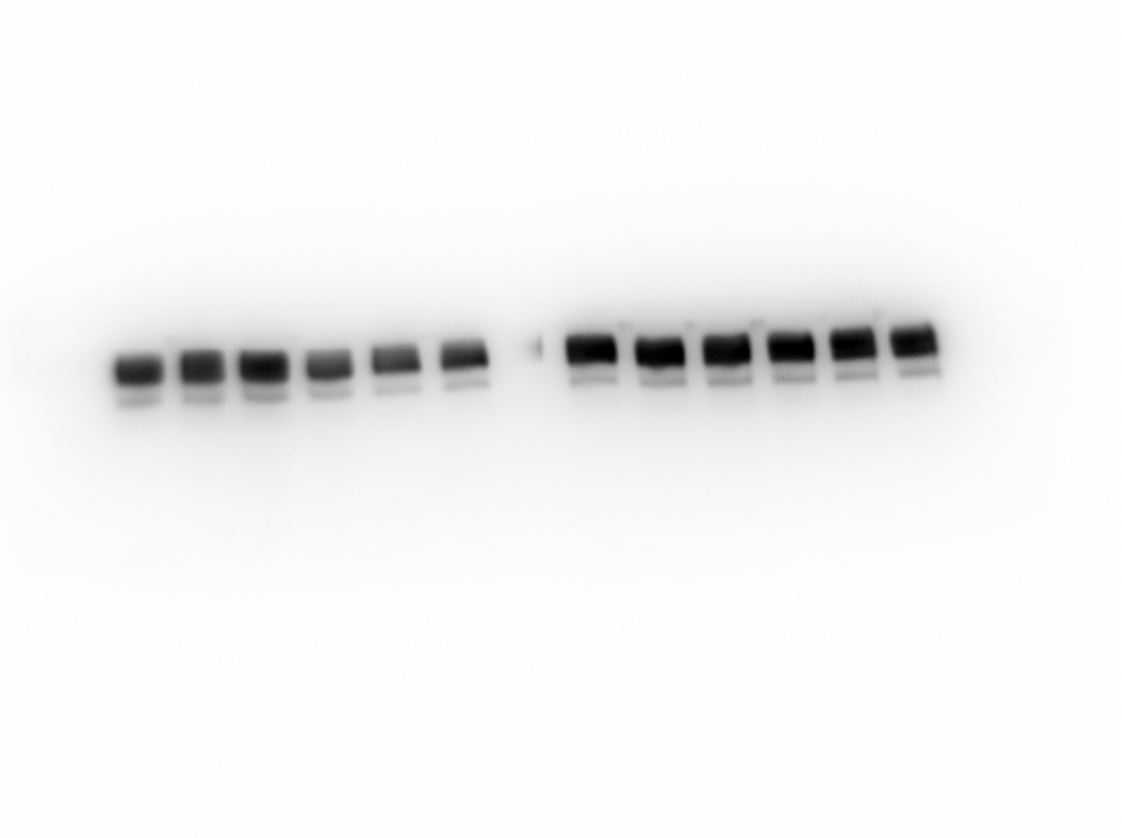

Supplement: Figure 9—figure supplement 1—source data 4. [file elife-73218-fig9-figsupp1-data4.tif]
